# Adult sex ratios: causes of variation and implications for animal and human societies

**DOI:** 10.1038/s42003-022-04223-w

**Published:** 2022-11-19

**Authors:** Ryan Schacht, Steven R. Beissinger, Claus Wedekind, Michael D. Jennions, Benjamin Geffroy, András Liker, Peter M. Kappeler, Franz J. Weissing, Karen L. Kramer, Therese Hesketh, Jérôme Boissier, Caroline Uggla, Mike Hollingshaus, Tamás Székely

**Affiliations:** 1 Department of Anthropology, East Carolina University, Greenville, NC USA; 2 Department of Environmental Science, Policy and Management and Museum of Vertebrate Zoology, University of California, Berkeley, CA 94720 USA; 3 Department of Ecology and Evolution, University of Lausanne, 1015 Lausanne, Switzerland; 4 Ecology & Evolution, Research School of Biology, The Australian National University, Acton, Canberra 2601 Australia; 5 MARBEC Univ Montpellier, CNRS, Ifremer, IRD, Montpellier, France; 6 ELKH-PE Evolutionary Ecology Research Group, University of Pannonia, 8210 Veszprém, Hungary; 7 Behavioural Ecology Research Group, Center for Natural Sciences, University of Pannonia, 8210 Veszprém, Hungary; 8 Behavioral Ecology and Sociobiology Unit, German Primate Center, Leibniz Institute of Primate Biology, 37077 Göttingen, Germany; 9 Department of Sociobiology/Anthropology, University of Göttingen, 37077 Göttingen, Germany; 10 Groningen Institute for Evolutionary Life Sciences, University of Groningen, 9747 AG Groningen, The Netherlands; 11 Department of Anthropology, University of Utah, Salt Lake City, UT USA; 12 Institute of Global Health, University College London, London, UK; 13 Centre for Global Health, Zhejiang University School of Medicine, Hangzhou, P.R. China; 14 IHPE Univ Perpignan Via Domitia, CNRS, Ifremer, Univ Montpellier, Perpignan, France; 15 Stockholm University Demography Unit, Sociology Department, Stockholm University, 106 91 Stockholm, Sweden; 16 Kem C. Gardner Policy Institute, David Eccles School of Business, University of Utah, Salt Lake City, UT USA; 17 Milner Centre for Evolution, University of Bath, Bath, BA2 7AY UK; 18 ELKH-DE Reproductive Strategies Research Group, Department of Zoology and Human Biology, University of Debrecen, H-4032 Debrecen, Hungary

**Keywords:** Behavioural ecology, Social evolution

## Abstract

Converging lines of inquiry from across the social and biological sciences target the adult sex ratio (ASR; the proportion of males in the adult population) as a fundamental population-level determinant of behavior. The ASR, which indicates the relative number of potential mates to competitors in a population, frames the selective arena for competition, mate choice, and social interactions. Here we review a growing literature, focusing on methodological developments that sharpen knowledge of the demographic variables underlying ASR variation, experiments that enhance understanding of the consequences of ASR imbalance across societies, and phylogenetic analyses that provide novel insights into social evolution. We additionally highlight areas where research advances are expected to make accelerating contributions across the social sciences, evolutionary biology, and biodiversity conservation.

## Abstract (web summary)

A detailed Review across animal and human societies provides insight on the causes and consequences of adult sex ratio skew.

## Introduction

The age and sex structures of populations across many animal species are currently shifting in response to anthropogenic impacts, including climate change and habitat loss^1–3^. Many human populations are also experiencing rapid demographic shifts as economic migrants, refugees, and other displaced people introduce population-level change to countries across the globe^4^. Together, these processes have transformed local adult sex ratios (ASRs) and generated substantial worry for societal issues (e.g., patterns of violence, family formation dynamics)^5^ and biodiversity conservation (e.g., population viability)^6,7^. While sex ratio skew is a topic of acute contemporary concern, it also has a deep history in the social and biological sciences. During the 19th century, early sociologists and naturalists noted imbalanced ASRs across a range of human and animal populations. For example, Du Bois’ pioneering work applying statistics to the social sciences identified the relationship between sex ratios and pair-bonding^8^. Follow-up work in the 20th century demonstrated ASR as an important population-level driver of reproductive behavior^9–11^, although this relationship remained largely understudied until relatively recently (see Box 1).

This knowledge gap is driven, in part, by the lack of interdisciplinary exchange across the social and biological sciences. As a result, insights have been slow to cross disciplinary boundaries. To achieve a more comprehensive understanding, what is needed is a conceptual, theoretical, and methodological integration of the processes that link the ASR to social behavior in both human and animal societies. This goal is important not only for disciplinary advancement, but also for practical applications in many fields—from the spread of diseases in human populations to the responses of wild populations to anthropogenic impacts.

Here we provide an overview of the current status, challenges, and prospects of ASR research, a multidisciplinary area that focuses on the causes and consequences of sex ratio variation among adult organisms. We begin the review with a description of the determinants of sex ratio variation across the lifetime of organisms and define key terms and concepts. We next shift to the consequences of ASR skew for a diverse array of behaviors related to reproduction, competition, investment, and social organization. We then review the literature on ASRs and their consequences across human societies to allow for comparative links to be made with animal systems. Finally, we conclude the review with a synthesis of the current state of the field, overview the main challenges that lie ahead, and offer future research directions and public policy insights.

Box 1 History of ASR research Darwin, in his 1871 book, *The Descent of Man and Selection in Relation to Sex*^90^, first highlighted the importance of ASR for sexual selection through the chapter entitled *Numerical proportion of the two sexes*. Darwin’s realization stemmed from a recognition that mating competition was affected by mate availability: *sexual selection would be a simple affair if the males were considerably more numerous than the females*. By pulling together an impressive amount of data from across both domesticated and wild animals, Darwin concluded that skewed ASRs are common and thought *a numerical preponderance of males would be eminently favorable to the action of sexual selection*.

During the 19th century, social scientists also noted the relevance of ASR for patterns of mating and parenting. In his seminal work, Du Bois^8^ offered influential insights for the role of partner availability on patterns of pair-bonding. Specifically, the results of his work from among African-Americans in the city of Philadelphia indicated that a shortage of men was associated with lower rates of marriage and higher rates of separation. Follow-up work in the early 20th century found similar patterning, with Groves and Ogburn^9^ arguing that relationship formation followed principles of an economic market. That is, the proportion of men to women influences their relative bargaining power and, therefore, willingness to marry, as well as the importance of various traits in a potential partner.

Parallel to this research being conducting in the social sciences, sex ratios were being evaluated in the biological sciences as well. For example, evolutionary biologist Mayr^10^ examined ASR variation across various species of birds. From this work, he contended that ASRs and mating systems were related. Specifically, monogamy was generally more common with an excess of males and polygyny with an excess of females. However, despite this early insight across disciplines, the causes and consequences of ASR variation largely remained unstudied until relatively recently^5,7^.

## Causes and implications of ASR variation

### ASR and its relationship to other sex ratios

Across a wide variety of dioecious animal systems (i.e., individuals produce either male or female gametes), researchers often assume that there is a near parity of males to females. While broadly accepted, this is an incorrect characterization of sexually reproducing organisms^6,7,12,13^. Though variable across species and populations, the ASR regularly deviates from 1:1. Measures of ASR in natural settings are most often derived from counts of live or dead individuals, either observed or captured. However, accurate estimates can be difficult to obtain and may be significantly affected by sex differences in behavior and conspicuousness that affect detectability. For example, among ungulates and primates, females are often group-living and are therefore more easily encountered (and counted) compared to males that are typically solitary^14,15^. Undercounting can also be of concern among sexually dimorphic species like songbirds, where males tend to have brighter plumage and more noticeable visual and vocal displays than females^16,17^. Therefore, to estimate the ASR accurately in wild populations, species and sex-specific detection probabilities need to be incorporated in the analyses (see Box 2).

**Fig. 1:**
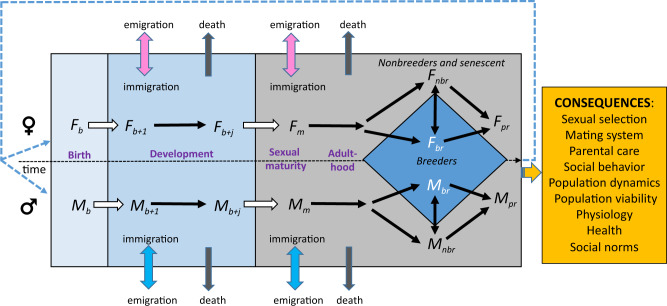
Sex ratios at various life stages and their consequences. Males (*M*) and females (*F*) flow through stages from birth (*b*) through development into juveniles (or subadults) for up to *j* time steps, maturation (*m*), and adulthood. Adults include newly mature individuals and individuals who reached sexual maturity at an earlier time. Adults are classified as breeders (*br*), nonbreeders (*nbr*) that are capable of breeding but at present are not reproductively active, and post-reproductive individuals (*pr*) that are senescent. Transitions between stages are shown with white arrows and within stages with black arrows. The number of females and males, respectively, are depicted at birth (*F*_*b*_ and *M*_*b*_), one (*F*_*b+1*_ and *M*_*b+1*_) and *j* time steps later (*F*_*b+j*_ and *M*_*b+j*_), at maturation (*F*_*m*_ and *M*_*m*_), breeding (*F*_*br*_ and *M*_*br*_), non-breeding (*F*_*nbr*_ and *M*_*nbr*_) and post-reproduction (*F*_*pr*_ and *M*_*pr*_). Different sex ratios emerge from various combination of the sexes at different stages: (1) Birth sex ratio = *M*_*b*_ / (*F*_*b*_ + *M*_*b*_); (2) Juvenile sex ratio = (*M*_*b+1*_ + *M*_*b+j*_) / (*F*_*b+1*_ + *F*_*b+j*_ + *M*_*b+1*_ + *M*_*b+j*_); (3) Maturation sex ratio (MSR) = *M*_*m*_ / (*F*_*m*_ + *M*_*m*_); and (4) Adult sex ratio (ASR) = (*M*_*m*_ + *M*_*nb*_ + *M*_*br*_ + *M*_*pr*_) / (*F*_*m*_ + *F*_*nb*_ + *F*_*br*_ + *F*_*pr*_ + *M*_*m*_ + *M*_*nb*_ + *M*_*br*_ + *M*_*pr*_). Consequences of sex ratios discussed in the paper are shown.

While the ASR is of central importance to population structure, it is but one of nine types of sex ratios measured across different ages/stages of development (e.g., fertilization, birth, and independence; Fig. 1)^18^. The ASR includes all individuals that have reached sexual maturity, regardless of sexual activity. Though often mistakenly used interchangeably with the operational sex ratio (OSR)^19,20^, the OSR actually refers to a *subset* of adults from the ASR who are currently available for mating. As such, it generally excludes sexually inactive, pregnant, and parenting adults^21^. Consequently, the OSR tends to be male-skewed in many mammals, because of the shorter receptive period of females compared to the duration of sexual activity among males, whereas the ASR is often female-skewed^6,22^. While the OSR is relevant conceptually to understanding sexual selection and breeding system evolution, identifying sexually active versus inactive animals in field studies is often challenging, thereby limiting its empirical use due to the inaccuracy of estimates^7,20,23,24^. Presently, the differential effects of ASR vs OSR on social behavior are not well understood and this is an active research area^7,20,25^.

An additional sex ratio to consider is the maturation sex ratio (MSR). The MSR is the ratio of males to females in a cohort that reach maturity (Fig. 1). Patterns of juvenile mortality, driven by, for example, sex-biased resource demands and predation rates, can push the MSR away from parity, thereby impacting mate availability^6,26^. An important consideration for sex ratio variability is that sex ratios at fertilization, birth, and independence are each characterized by a single cohort and, due to their smaller sizes, are likely more variable than the ASR which is usually inclusive of multiple cohorts (Fig. 1).

Another challenge, specific to human studies, is that the ASR is variably defined within and among societies (Box 2)^5^. This occurs because the definition of who is an ‘adult’ is often defined by cultural, religious, and legal norms, suggesting that ASR calculations for humans are not simply based on sexual maturation. Nevertheless, men and women usually reach adult status by their late teens across societies—both culturally and biologically^27^.

Box 2 Quantifying adult sex ratio The ASR is one of the fundamental characteristics of populations, and producing valid estimates requires defining the ages inclusive of adulthood, determining which ratio estimator to use, and deriving a measure that accounts for differences in detectability between the sexes. Intuitively, ASR includes all individuals at (and beyond) the age of sexual maturation, whether or not they are currently sexually active. However, in human societies, sexual maturity often occurs years before societies traditionally or legally bestow the privileges and responsibilities of adulthood^5^. In wild populations of dioecious animals (i.e., individuals produce either male or female gametes), the ASR is typically comprised of all reproductive and non-reproductive individuals that have reached sexual maturation, and generally includes post-reproductive individuals as well^18^. Including the non-reproductive individuals in ASR estimates is justified for two main reasons. First, distinguishing reproductive vs non-reproductive individuals is not straightforward in most populations, including humans, since sexual activity can be difficult to detect. Second, while some individuals may not be currently reproductively active—for instance they are unable to secure a mate—their presence pressures mating and parenting decisions for both same and opposite sex individuals^20,105^.

Sex ratios can be expressed in numerous ways, including the number of males per female and the number of males per 100 females on arithmetic or log scales. However, ratio-based estimators of ASR are problematic because they are asymmetric, being bounded by zero on one end and unbounded at the other end (i.e., positive infinity). We propose to express ASR as the proportion of males in the adult population [ASR = N_males_/(N_males_ + N_females_)]. It is easy to interpret this measure since it is bounded between 0 (only females in the population) and 1 (only males), it reflects the relative abundances of males and females in the adult population, and it is easy to convert to percentage of males in the adult population.

Accurately quantifying the ASR requires unbiased estimates of the number of adult males and females. An ASR is typically estimated from live or dead individuals that are counted or captured. However, count-based estimates can be affected by sex differences in behavior or conspicuousness. Males and females often exhibit different habitat preferences, differ in daily and seasonal activities and possess different body sizes, coloration and weaponry, and these sex differences could bias male and female encounter rates^53,54^

Fortunately, recent statistical advances have developed estimators that account for detection error in the counts of unmarked individuals (observed, trapped or killed) and counts of marked individuals^171–173^. For example, Ancona et al.^18^ illustrate how sex-specific detection *(p*), estimated from mark-recapture models, can be used to estimate sex-specific population sizes (*N*) to estimate the ASR, where ASR = (*N*_male_ /*p*_male_)/(*N*_male_ /*p*_male_ + *N*_female_ /*p*_female_).

The ASR in humans is sometimes called the population sex ratio and typically is estimated from census data^155^. Although these data are often of good quality, they are not free of errors^174^. Censuses may miss or double-count one sex more frequently than the other. This can occur when migration rates, privacy concerns, or misreporting of age differs between men and women, and can bias sex ratio estimates^175^.

### How do biased ASRs emerge?

ASR is a demographic property of a population that is initially driven by sex ratios at conception and birth, and further altered by sex differences in rates of maturation, mortality, dispersal, and immigration^28,29^. Therefore, skewed sex ratios result from differences in these processes across various life stages including (i) pre-birth/birth, (ii) juvenility and subadulthood, and (iii) adulthood (Fig. 1, Box 3).

(i) There are multiple mechanisms that can bias sex ratios at conception or at birth, including diverse sex determination systems (see below), various selfish genetic elements that enhance their own transmission^30^, and microorganisms (e.g., the bacteria *Wolbachia* targets and kills male (or female) embryos shortly after conception)^31^. Sex determination systems may produce strongly skewed offspring sex ratios, given that sex determination can be genetic (GSD), environmental (ESD), or some combination of the two (“environmental sex reversal”; ESR). Environmental factors that can influence sex determination are diverse and include ambient temperature, pollutants (especially endocrine-disrupting chemicals), pH, aspects of the social environment, and water availability^32^. While the mechanisms are not entirely clear, many of the aforementioned factors appear to induce physiological stress that affects sex determination^33,34^, possibly through the modulation of energy balance^35^.

Environmental influences on sex allocation have been observed in many invertebrates, fishes, and reptiles^35–39^. Among fish with temperature-driven sex determination, higher temperatures typically lead to a male bias (ESD) or to masculinization of genetic females under the influence of the environment (ESR), which generally occurs post-hatching^40,41^. Counterintuitively, though, rising temperatures from climate change may not necessarily result in more males. For example, changing temperatures can shift spawning times, thereby resulting in *colder* temperatures at the time of sex determination. Accordingly, for some species, climate change may result in more males in some locales and more females in others, thereby highlighting the need to target the local ecological context experienced by individual populations when assessing impacts of climate change^1^. Thermal fluctuations that accompany climate change are particularly concerning for endangered animals with ESD, such as tuatara and crocodilians, due to possible population collapse as a result of sex ratio skew^42^. In turn, shifting sex ratios, such as in marine turtles, where raising temperatures are producing more females, could have knock-on effects for reproductive skew and competition for mates among both males and females^25,43^.

(ii) Male and female juveniles and subadults may have different sensitivities to environmental stressors, such as food and diseases. Sex-biased juvenile mortality may reflect sex-specific life histories that affect, for example, the growth and timing of gonad formation^44^ or dispersal patterns^28,45–47^. In red deer, food shortage is especially harmful for young males due to their elevated caloric needs as a result of sexual dimorphism^47^. This relationship is also observed among bird species with sexual dimorphism, whereby the larger sex tends to die at higher rates due to resource scarcity^48^.

(iii) In many organisms, ASR skew only emerges during adulthood as a result of sex differences in adult survival^6,7^ due to ecology, life-history, and behavior. For instance, sex differences in the susceptibility of adults to predators, parasites, and diseases^49–52^ can lead to skewed ASRs, as well as sex differences in body size, behavior, ornaments, and armaments^53,54^. Sex differences in adult survival also persist in captive populations in the absence of predators and with abundant food^55^, suggesting a genetic basis to these differences^56^.

**Fig. 2:**
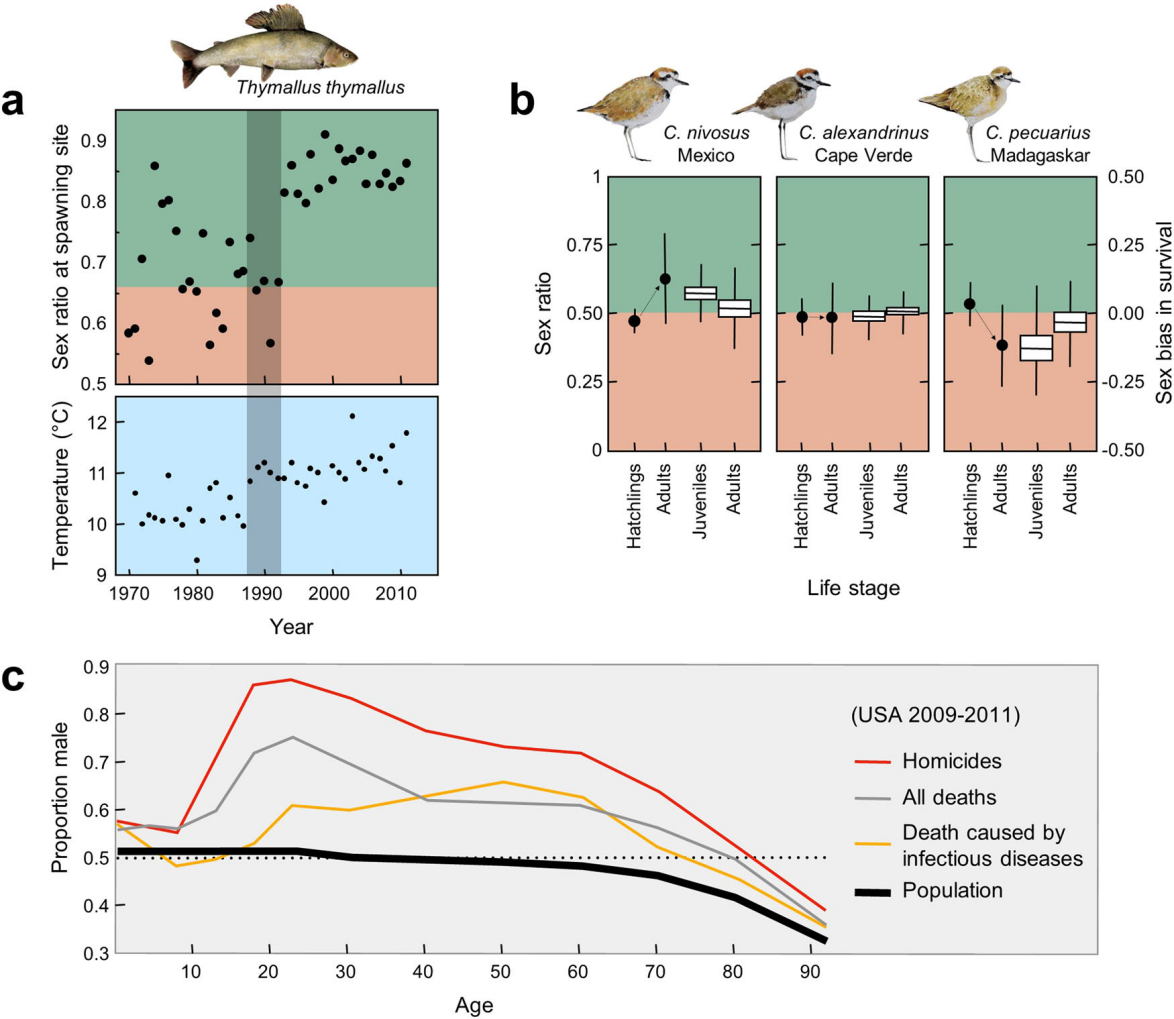
Causes of ASR variation in grayling, shorebirds, and humans. **a** Adult sex ratios link to climate change in grayling of Lake Thun, Switzerland: male-biased adult sex ratios during spawning period^57^ and average yearly water temperatures at the spawning site. The transition from the red to the green background indicates the average yearly adult sex ratio from 1948 to 1992. These adults were on average five years old, and the gray shading highlights the 5-year period after the global temperature regime shift in 1987/88^168^. **b** ASR and demographic parameters in three plover species (*Charadrius spp*): hatchling and adult sex ratios (round symbols; means and 95% CI) and sex-specific juvenile and adult survival (medians, quartiles, and ranges)^58^. **c** Sex ratios and proportions of death by sex across human age groups presented for homicides, infections/parasitic deaths, and all causes (US Census data 2009–2011). Drawings by Lara Wedekind using data from refs. ^58,168^.

Accordingly, these different processes act together from pre-birth and early development through adulthood to drive sex ratio variation across the life of an organism (Fig. 2a, b). For instance, detailed monitoring of graylings, shorebirds, and green-rumped parrotlets reveal ASR biases not driven by any single source, but instead by a combination of demographic factors^28,57,58^. The same is true for humans who, despite averaging a slightly male-biased birth sex ratio (51% male), experience considerable heterogeneity across the lifecourse in sex-biased mortality due to biological, environmental, and cultural factors^59–63^. Even so, male mortalities are regularly higher than female mortalities for all age groups and societies, resulting in, on balance, an eventual reversal of the birth sex ratio bias in later adulthood (Fig. 2c)^64^. An additional consideration, relevant across animal taxa, is that sex-biased dispersal of juveniles and/or migration of adults modulate locally driven demographic patterns, further influencing the ASR^12,28,65^.

For a comprehensive understanding of the causes of skewed ASRs, researchers need to (i) identify the sex ratio implications of sex determination systems under ecologically relevant conditions^66^, (ii) estimate sex-specific survival of embryo, juvenile, and adult stages^67,68^ (iii) examine sex-specific life histories that may affect growth, and the timing of maturation^69^, (iv) combine these demographic components into data driven models^28,58^, and (iv) integrate the demographic models with sex difference in movements of juveniles and/or adults.

Box 3 Extraordinary adult sex ratios Heavily skewed adult sex ratios can be the product of biased offspring sex ratios that persist into adulthood. For example, female-biased offspring production in various insects due to haplodiploid sex determination or sex-killing bacteria (e.g., *Wolbachia*, *Rickettsia*) can lead to spectacularly biased ASRs including *Hypolimnas* butterflies where over 90% of adults are females^176^. In species with environmental sex determination, warmer incubation temperatures can shift birth sex ratios to over 90% female in marine turtles, a sex ratio bias that is maintained into adulthood^177^.

Sex differences in mortality of juveniles and/or adults may also swing the ASR into extremes^54^. High mortality of adult males produces extremely female-biased ASRs in marsupials and external parasites such as lice and fleas^47,178^. In the brown antechinus, all males die after mating, apparently due to exhaustion and stress, so that the adult population consists of gestating or nursing females for about 7 months until the young males mature and are ready to mate^179^. Female-skewed ASRs appear to emerge via chemical exposure in lice and fleas because males die at higher rates following contact than do females. For instance, in the feather lice *Quadraceps aethereus*, over 95% of adults are females likely due to toxins produced by host seabirds that are deadly to male lice^180^.

Alternatively, high mortality of adult females may create heavily male-biased ASRs. The ASR of an island population of Hermann’s turtle is over 90% male. A major contributing factor to the extreme ASR is excess female mortality due to male harassment and sexual coercion resulting in female injury and death^181^. Schistosome internal parasites also exhibit male-biased ASRs that are largely due to their sexual size dimorphism emerging from different lifestyles of males and females, and from their monogamous mating system^182^. They live in blood vessels whereby the large muscular males are better able to resist blood flow than the less muscular females. Females instead live inside the groove of the male’s body^183^. A consequence of sexual dimorphism is the loss of many juvenile females because they are unable to resist against the flow of blood during their development. Female schistosome parasites capitalize on the male-skewed ASRs by frequently changing partners, since mate change by females is about three times more common than mate change by males as shown by experimental manipulation of ASR^183^.

**Box 3 Fig: The influence of experimentally manipulated adult sex ratio (ASR) on divorce rates in** ***Schistosoma mansoni*** **parasites**^148^. The divorce rate is positively correlated with ASR when it is male-skewed (in blue) but not female-skewed (in pink). The size of the circles is proportional to the number of pairs, which varies from 2 to 10. The inset shows a *Schistosoma* pair with the muscular male hosting the slender female in his ventral groove.
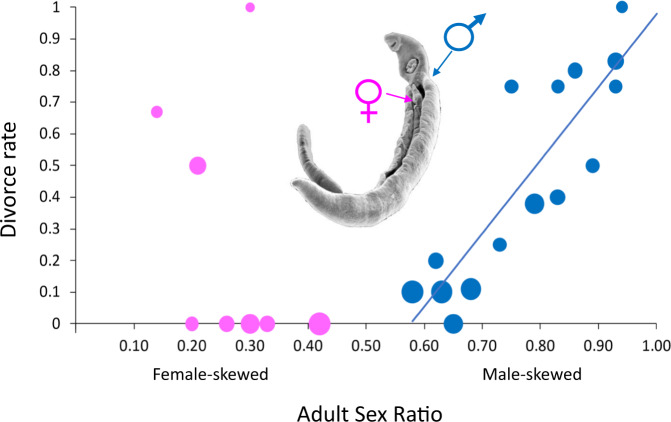

### Condition dependent sex determination and sex change

**Fig. 3:**
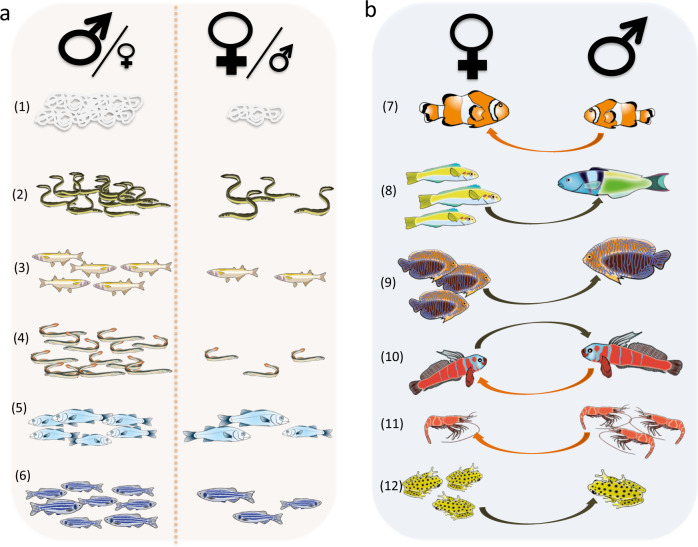
Condition-dependent sex determination and sex change. **a** Density-dependent sex determination potentially affecting ASR in (1) the nematode *Romanomermis culicivorax*, (2) temperate eels, (3) the pejerrey, (4) the brook lamprey, (5) the European sea bass and (6) the zebrafish *Danio rerio*. In all the above-mentioned species, more males are produced at high density. **b** Socially induced sex change occurs in various species such as (7) protandrous clownfishes, protogynous (8) wrasses (e.g., *Thalassoma bifasciatum*) and (9) Potter’s angelfish as-well as bi-directional sex change as exemplified in (10) the blue-banded goby. Other examples of socially controlled sex change were observed in both crustaceans and amphibians: (11) Northern shrimp exhibit protandrous sex change that occurs at small size when the density of females in the population is high. Protogynous sex change was also observed in (12) captive reed frogs and its occurrence is linked to local male density. Hence, for most sex changing species, those individuals that do not change sex are more numerous. Note that the direction of the arrow in the right panel (**b**) indicates the direction of sex change: orange from male to female (protandrous) and maroon from female to male (protogynous). Drawing by Pierre Lopez (MARBEC) based on data from refs. ^71–82,169^.

The social context experienced by an individual can induce them to facultatively develop into a given sex. The ASR is involved in this process as both a cause and consequence. Specifically, sex determination and sex change are variably influenced by either encounter rates of males vs. females or local population density (Fig. 3a, b).

#### Sex determination

In the green spoon worm, sex determination of the larvae depends on the local ASR or, more precisely, on the individual they first encounter: larvae develop either as a female if they first find an empty burrow or male if they first encounter a female. This is driven, in part, because it is the male who lives within the female, and thus secures a future partner through this process^70^. In terms of population density, crowding, in most cases, results in an ASR bias in favor of males (Fig. 3a). Sex determination of the nematode *Romanomermis culicivorax* is density dependent and is biased toward females at low density and males at high density (Fig. 3a)^71^. Population density also correlates with masculinization so that high density leads to male-biased ASRs among a variety of fish species including temperate eels (American, Japanese and European eels), pejerrey, lampreys, European sea bass, and zebrafish (Fig. 3a)^72–77^. Mechanistically, stress and masculinization in the above-described situations are linked because more males are produced in relatively harsher biotic conditions^33^. In eels, zebrafish, and pejerrey, cortisol (the major stress hormone) was proposed as a main contributing factor to masculinization at high density, as was the temperature^74–76^.

#### Sex change

Socially induced sex change has been observed in crustaceans, fishes, and amphibians (Fig. 3b). The local ASR can induce sex change in hermaphroditic species, from male to female (protandry), as exemplified in various species of clownfish where males change sex when the biggest individual (female) dies or emigrates (Fig. 3b)^78^. Conversely, sex change can also take place from female to male (protogyny). This often occurs among fish where a single male controls a harem of females but, when his dominance wanes, a female subordinate changes sex (e.g., bluehead wrasse; Fig. 3b)^79^. Particularly striking examples of ASR-induced sex change comes from gobies, where individuals can adaptively change sex in either direction, depending on the sex that they interact with most (Fig. 3a)^80–82^.

Activation of the stress-axis has been identified as a main driver of sex change across protogynous species, where social interactions are crucial in determining both the dominance hierarchy and sex^79,83,84^. In these species, a rapid increase of cortisol in the dominant female triggers masculinization. Mechanistically, cortisol can inhibit the production of aromatase (i.e., the enzyme responsible for the conversion of androgens into estrogens) and promote testes development^83^. In protandrous clownfish, the dominance of females over males is persistent and cortisol is also suspected to be involved in the maintenance of the males’ testes^85^. In large groups (characteristic of most protogynous species), dominants were found to be more stressed than in small groups (characteristic of most protandrous species), where subordinates were generally more stressed than dominants^86^. This pattern likely explains why the same mechanism, involving stress, would be involved in distinct sex-change strategies (protogyny vs protandry).

### ASR, mating competition, and parental care

**Fig. 4:**
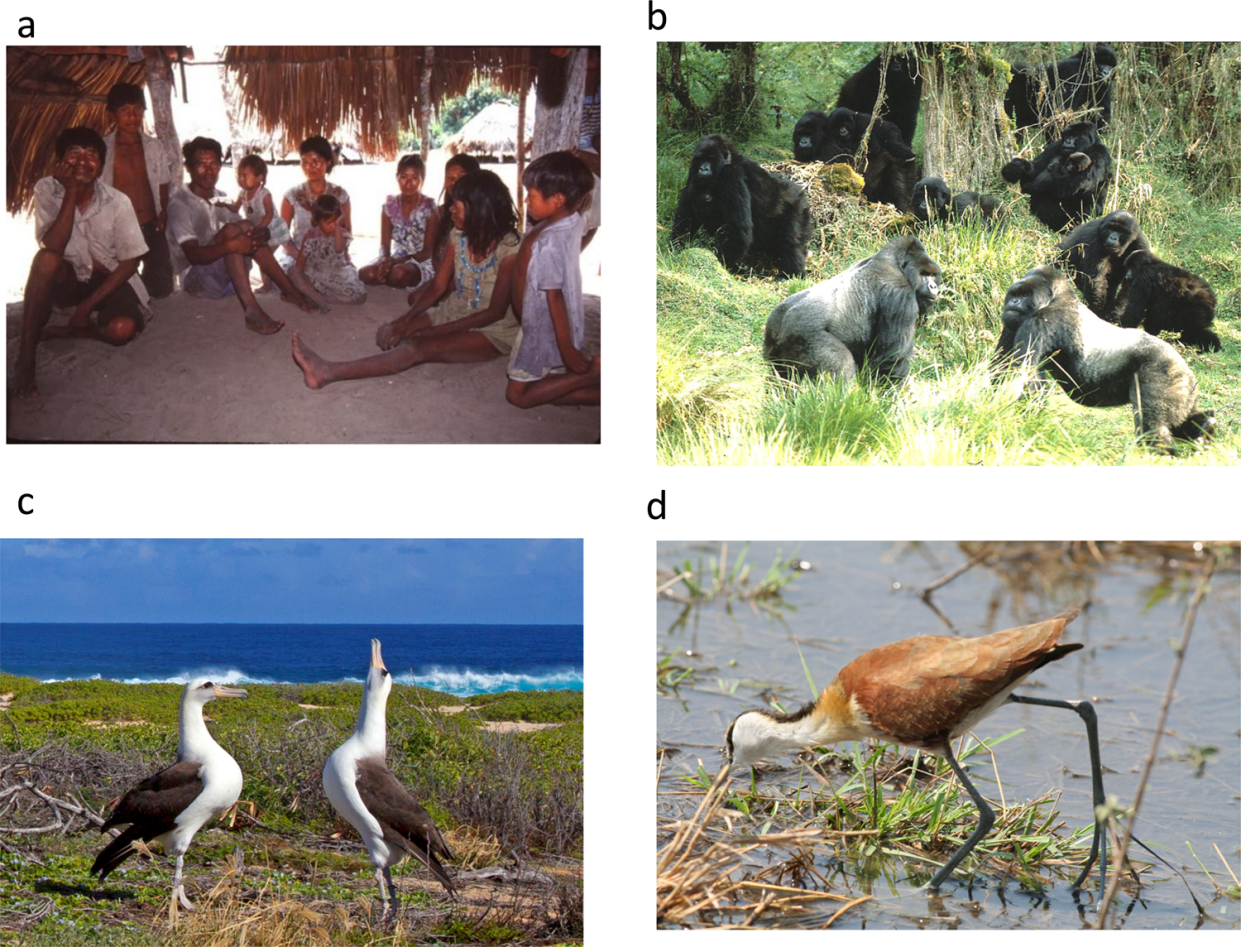
Adult sex ratio variation and its implications for mating systems. **a** Small populations, such as human hunter-gatherers, are particularly susceptible to variation in partner availability which can result in flexible, yet fragile, pair-bonds (e.g., Savanna Pumé, credit: R.D. Greaves)^12^; **b** polygyny and male size dimorphism are common among species with female excess (e.g., mountain gorilla, credit: A. H. Harcourt)^47^; **c** monogamy and biparental care are characteristic of even sex ratios and slight male excess across many species (e.g., Laysan albatross, credit: A. Badyaev)^111^; **d** as male-bias in the adult sex ratio becomes even more dramatic, polyandry, female-biased sexual dimorphism and sex-role reversal are common (e.g., African jacana, credit: T. Székely)^110^.

To reproduce successfully, dioecious organisms may need to pass through several major stages of reproduction: find a mate, decide whether to divorce or keep the mate for future breeding, and provide care for the young if necessary (Fig. 4a–d). The relative frequencies of adult males and females are expected to fundamentally structure behavior across these stages, particularly because the rarer sex in a population is expected to have greater bargaining power in mate choice, pair bonding, and parenting decisions^5–7,87^. In addition to the interactions between males and females in the context of reproduction, ASR can also influence male-male and female-female interactions. Experimental and comparative studies regularly support these expectations, although recent work highlights the need to explore more diverse interactions responsible for a suite of subtle fitness implications^88,89^.

#### Mate choice

Darwin^90^ thought that sexual selection should be a straightforward process whereby males, to secure a mating, compete more intensively with increasingly abundant males. Recent work, however, provides a more nuanced view of mating behavior with respect to the ASR. Specifically, mating rates at male-skewed ASRs tend to *decrease* for males and *increase* for females^91^. For example, while male courtship rates do indeed increase with male-skewed ASR in fruit flies and gobies^92,93^, their success rates decline. In addition, females become increasingly choosy and spend more time discriminating between individual males when more males are available to mate^91,92,94^.

Consequently, ASR fluctuations in wild populations may trigger complex and facultative mate choice decisions. For example, among Darwin’s finches, the choice of a mate is generally influenced by learning through early experience. However, fluctuations in environmental quality that drive sex ratio skew have more immediate consequences for mate choice^95^. Specifically, in years of drought, a male-bias emerges resulting in fewer extra-pair mating opportunities for males and greater choosiness among females. Females additionally benefit from male-biased sex ratios through more frequent polyandry and elevated fertility than is observed at even sex ratios^95^.

Importantly, with shifts in the ASR, not only does the number of competitors change but so does the reward for successful individuals^96,97^. For instance, with increasingly female-skewed populations, the average number of mates per male increases. Consequently, variance in male mating success also increases due some males being better able to monopolize sexual access to females^5^. Thus, because sexual selection is linked to variance in reproductive success, mating skew (and therefore sexual selection) becomes amplified for males in populations with female-skewed ASRs. A recent phylogenetic analysis provides empirical support, showing that male-biased sexual size dimorphism (an often-used indicator of sexual selection) in birds and mammals is most pronounced not in species that exhibit male-skewed ASRs, as Darwin conjectured, but rather in female-skewed species^98^, an outcome consistent with early studies of sexual size dimorphism and sex ratios in both birds and mammals^99,100^.

Population density, however, may modify male and female mating strategies and the response of individuals towards skewed ASRs. In fruit flies and beetles, fertilization success increases with both male-skewed ASRs and population density^101^. The effects of the ASR and density, however, is not simply additive because fertilization success increases more quickly with male-skewed ASR at high population densities than at low densities. Accordingly, intense sexual selection (i.e., high reproductive skew, intense competition, and high risk of being left out of mating among males) may be observed at male-skewed ASRs with high population densities^102^. Importantly, results from lab-based studies are consistent with field studies across a variety of species^103,104^.

#### Mating systems and pair bonds

The number and distribution of mates, and the duration of pair bonds vary substantially among animals, and recent works highlight the significance of ASR in their variation. The excess of either males or females in a population can affect fitness payoffs for species-specific patterns of pair-bonding. Enhanced mating opportunities for the rarer sex in the population appears to destabilize monogamous pair-bonds and lead to multi-mate families and/or divorce to find new partners^20,91,105^. Studies of wild populations with flexible breeding systems tend to be consistent with these theoretical predictions^54^. In populations which exhibit variation in their ASR, polygyny by males and polyandry by females were associated with female-skewed or male-skewed ASR, respectively, suggesting that changes on ecological time scales in relative frequencies of the sexes could variably favor one sex over the other in terms of payoffs to multiple matings^106–108^.

Comparative studies of evolutionary time scales across different species additionally support these findings. Polygyny by males is associated with female-skewed ASRs, whereas polyandry by females is usually associated with male-biased ASRs^109,110^. However, an unresolved question from these studies is whether species exposed to long-term ASR bias across evolutionary time will also be responsive to increasing temporal and spatial variation in ASR at an ecological time scale due to rapidly changing environments.

#### Parental care

Theoretically, a surplus of mating partners can entice a parent to abandon their family and start a new reproductive event with a different mate (Box 4). Thus, in species that exhibit multiple types of caring within a population (e.g., female-only care, male-only care, biparental care), females may abandon their families at male-skewed ASR whereas male abandonment is more common at female-skewed ASR^105,111^. However, payoffs to novel mating opportunities in response to ASR skew can be highly variable, even among closely related species^112^. Specifically, the relative parenting roles of males and females make reliance on care a key driving consideration for benefits to the pursuit of additional mating opportunities^58,113^. The responses of individuals in flexible social systems on ecological time frames are consistent with broad scale phylogenetic analyses where male-skewed ASRs are associated with more care by males relative to females on evolutionary time scales, at least in birds^109^.

Two factors prohibit drawing strong conclusions from past studies. First, untested is the assumption that demographic and behavioral estimates from current populations are robust to temporal and/or spatial variation^28,95^. Because fluctuations in these traits are common, demographic variation could undermine broad phylogenetic associations. Nevertheless, ASR estimates are often consistent between different temporal and/or spatial estimates in birds and mammals^18^. These studies suggest that ASR estimates, at least in contemporary populations, are reliable. Second, the responses of parents to ASR variability at ecological time scales are limited by the plasticity of their parenting traits. Parents of some species have specific adaptations to providing care, such as a brood pouch in male pipefishes or mammary glands in female mammals, which can constrain responsiveness to ASR skew because offspring survival depends on a caring parent possessing these specific traits^114^.

#### Sex roles and sex ratios

Although the evolution of sex roles is a contentious subject, evolutionary associations between sex ratios and sex-biased patterns of care and competition are proposed to have emerged via one of two routes. First, due to sex differences in mortality, a skew in the ASR could have emerged in a population and, via frequency-dependent selection, resulted in sex-biased payoffs to mating, pair-bonding, and parenting behavior for males and females^7,115^. Second, early in the course of evolution, sex differences in gametic investment may have led to sex differences in costs to parental care and biases in the OSR (i.e., who is available to mate)^97^. The downstream consequences could have been more intense competition and amplified expression of traits involved in sexual selection in the non-caring sex (e.g., weaponry, gaudy coloration), thereby elevating the mortality risk of the bearer and resulting in ASR bias^11,21^(T. H. Clutton-Brock pers comm). Both scenarios have theoretical support, yet more work is needed to establish which one best fits a particular group of organisms^15,98^.

Box 4 Effects of sex ratios on parental sex roles In most species with parental care, parents differ in the amount of care they provide to their offspring. If egalitarian biparental care is not required for offspring survival and development, there is a conflict of interest on the amount of care to be provided by the male and female parent. The ‘Fisher condition’^184,185^ is crucial for predicting the outcome of this conflict. In sexually reproducing diploid species, each offspring has one father and one mother. Hence, the total reproductive output of all adult males must exactly match the total reproductive output of all adult females. Any bias in ASR has a straightforward implication: if one sex is k > 1 times more abundant than the other, a member of the minority sex produces, on average, k times as many offspring as a member of the majority sex. If the ASR of a cohort is fully determined at the time of maturation and does not change later in life, a straightforward line of argumentation^20,186^ reveals that the majority sex in adulthood is selected to do most of the caring, assuming that parenting roles are evolutionarily flexible. Hence, a male-biased ASR is predicted to lead to male-biased care, while a female-biased ASR leads to female-biased care.

This simple causality breaks down if the ASR of a cohort is not constant but affected by differential mortality between the mating stage and the caring stage, and/or differential mortality between the sexes^187,188^. In this case, the source of ASR variation matters. For example, if the sex ratio at maturation is 1:1 and the sexes differ in mortality at the caring stage, the sex with the lowest care-mortality will be selected to do most of the caring^188^. If care-mortality is substantial, the ASR will become biased toward the non-caring sex (opposite to the standard expectation), as this sex avoids an important source of mortality. The most complicated situation arises when the sexes differ in mortality at the mating stage. As shown in a simulation study^188^, the same mortality pattern can lead to the evolution of either male-biased care or female-biased care. Again, the ASR will become biased toward the non-caring sex, in contrast to the standard expectation. The latter example shows that the same demographic parameters (i.e., sex-specific mortalities) can lead to alternative evolutionary outcomes, which differ in their care pattern and the resulting ASR bias.

The discussion above considers the adult sex ratio, but the sex ratio at conception (the ‘primary sex ratio’, PSR) and the sex ratio at the end of parental care (the ‘fledging sex ratio’, FSR) are also intimately related to parental sex roles. This is perhaps surprising, as Fisher’s Equal Allocation Principle^184,189,190^, which predicts a 1:1 sex ratio at independence of young^191^, seems to hold under quite general conditions. However, a recent simulation study^188^ shows that the joint evolution of the primary sex ratio and sex-specific care leads to parental sex roles in a predictable manner: if one type of offspring is ‘cheaper’ in that it has a lower mortality or requires less parental care, the sex ratio of young at independence should not be 1:1 but biased to the cheaper sex and, all other things being equal, the cheaper sex at birth should do most of the caring when a parent.

All the above predictions consider relatively simple scenarios with few feedbacks between different mortality implications of reproductive behaviors (“all other things being equal…”). Simulations indicate^188^ that even under these conditions parental sex roles can be ‘evolutionarily labile’ in that they readily switch between alternative equilibria. This does not change if factors like sexual selection are added to the model: for the same parameters there are alternative evolutionary outcomes, and sexual selection, sex ratios, and parental care patterns affect each other in intricate ways. These theoretical insights—consistent with empirical studies—further bolster the need for advancing ASR as a multidisciplinary research program.

### Population dynamics and viability

The relative frequency of the sexes influences population growth and persistence over time^116^. For example, a female-skewed population is expected to grow faster due to potentially higher birth rates than a population of equal size with a male-skewed ASR. However, the relationships between ASR and population growth and viability are complex, and dependent on a variety of social characteristics, including mating system and group structure^115,117,118^. In general, mathematical models forecast that species with monogamous mating systems will have both high population growth rates and low extinction risks at balanced ASRs, whereas polygynous species are least vulnerable to extinction at female-biased ASRs. In addition, the ASR usually has a larger effect on population growth and persistence among polygynous rather than monogamous populations^115,117^.

Empirical studies corroborate the predicted influence of ASRs on fertility and population growth rates because male-skewed ASRs are associated with reduced female fertility and stationary (or declining) populations^45,119^. Reduced fertility and suppressed population growth can be driven by excess males and by physiological responses among females to the overabundance of males: high rates of male aggression elevate females’ stress levels, reduce their fecundity, and increase female mortality^120,121^. Experiments using common lizards indicate that females in their reproductive prime have higher reproductive success in female-skewed rather than in male-skewed populations, likely due to the reduced harassment by males^122^. To avoid male-driven mating aggression, females may migrate out of male-skewed populations and seek populations with more favorable ASRs^123,124^.

While females can potentially achieve high reproductive success in female-skewed populations, the outcome is often context-dependent^122,125,126^. Scarcity of males may limit female fertility, so that females may not find a mate, or the males they find are of poor quality and/or not able to fertilize all of their eggs^127–129^. In addition, because female fertilization success is influenced by the ASR, population density, and their interaction^101^, the effects of ASR on population viability also depend on population density. For instance, the scarcity of males at low population density is associated with reduced fecundity in first-time breeder female moose because of the limited ability of males to fertilize all of the females across expansive ranges^129^. Conversely, male excess can improve female fertility in monogamous species and allow females to select from a broader range of males and thus produce more viable offspring^130^. Furthermore, in species where males provide parental care, an excess of males increases female reproductive success through elevated resource provisioning by individual males;^106^ therefore, a surfeit of females could actually reduce population-level reproductive rates^131,132^.

Importantly, because a skewed ASR impacts the reproductive success and viability of populations, wildlife managers, conservation biologists, and captive breeders need to determine the optimal ASR for harvesting and/or conserving wild populations, and managing captive breeding stocks^133,134^. Firstly, the effective population size of many wild animals is substantially smaller than the total population size, partly due to skewed ASRs^135^. Therefore, preserving enough genetic variation in small populations with skewed and/or fluctuating ASRs will be especially challenging^45,136^. Secondly, selective removal of males for trophy hunting may disrupt social systems and counterintuitively, reduce harvestable returns^137^. A detailed understanding of social systems and demography is thus essential for optimal harvest and population management^132,137^. Thirdly, skewed adult sex ratios can have far reaching implications for threatened animals, as evidenced by the critically endangered Tasmanian parrot. An introduced predator primarily kills nesting females and their clutches, resulting in the remaining females mating with multiple males. Mixed paternity, however, results in low nesting success and population viability models predict steep population declines^138^. Taken together, understanding the causes of skewed ASRs and their implications are not only essential for advancing evolutionary biology, but also significant for real life biological applications^18,45,47,119,123,139^.

### ASR effects in space and time

**Fig. 5:**
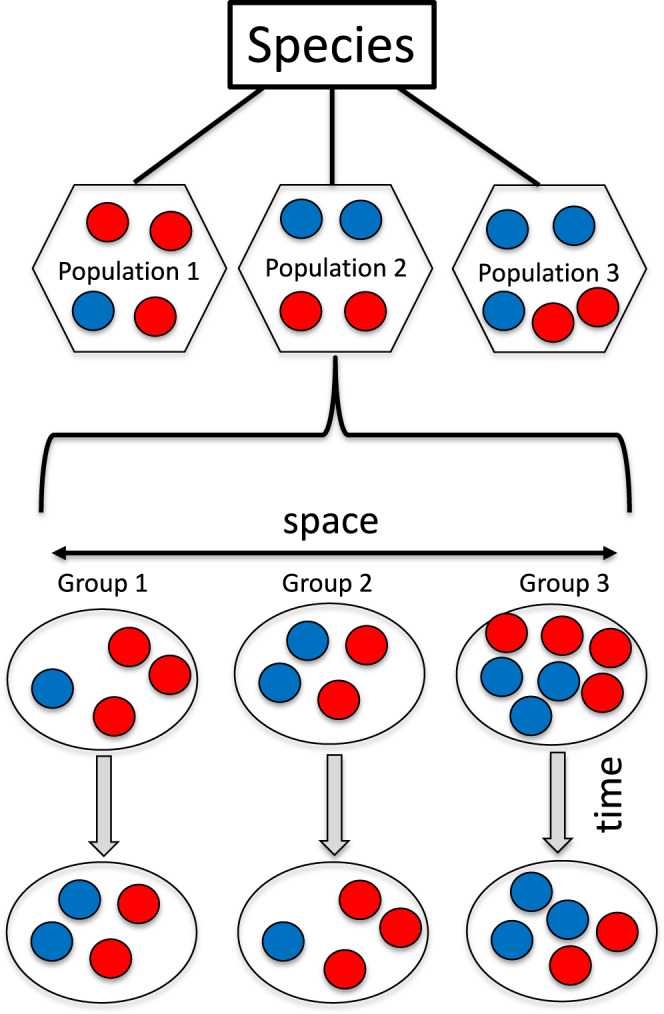
Spatial and temporal variation in ASR. Every social group may have different number of adult females (red circles) and males (blue circles). A group’s ASR will change as a result of deaths, maturations, emigrations and immigrations over time, and neighboring groups of a population often have different ASRs. Adults may move between groups, and local ASR may trigger these movements^120,170^. The average ASR may vary across populations of the same species. This variation also raises the questions whether the current local or the average long-term population- or species-specific ASR underly variation in social behavior and how animals perceive the relevant sex ratio.

Interspecific variation in ASR is large across animal species, ranging from a strong female bias in isopods (only 1% of individuals are males) to a strong male bias in some reptiles and birds (90% male; Box 3)^54^. With this variability, broad-scale analyses on the effects of ASR on mate acquisition, mating systems, and parenting has focused on correlations between the average ASR of a species and one, or several, components of social or reproductive behavior. For example, ASR is associated with mating systems and sexual size dimorphism;^98,110^ the latter is a useful index of the intensity of male-male competition and has been shown to increase with progressively female-biased ASRs^98–100^. While these and other studies revealed new insights about evolutionary associations between sex ratios and sexual selection, such a comparative approach overlooks variation among individuals and populations. It further raises fundamental questions about the level of population aggregation, mechanisms, and plasticity of social behavior in the face of ASR variation (Fig. 5).

Long-term field studies reveal substantial intraspecific variation in ASR over time^7,14,57,95^. For example, the ASR in a marsupial population changed more than two-fold over just five years^140^, and field studies among fish have revealed even more pronounced fluctuations across a single breeding season^92^. In humans, warfare results in punctuated male-biased mortality events that can dramatically shift ASRs over short time scales, in both small-scale and industrial societies^141,142^. The ASR can additionally vary spatially, either among different populations or among neighboring social units, as in many non-human primates, humans, and mice^12,143^. Hence, a biologically meaningful measure of ASR for breeding systems and social behavior may vary. For some species it may simply be the ASR of the immediate group, while in others it may be the ASR of the wider neighborhood, inclusive of other groups and potential floater individuals. Nonetheless, while ASR may vary temporally or spatially, reactions to variability in partner availability may be phylogenetically constrained due to, for example, aspects of mating system and social organization. Behavioral options to fluctuating ASR are likely more limited among pair-living birds (due to offspring bi-parental care demands) than group-living mammals.

The observed plasticity in mate choice, pair-bonding, and parenting gives rise to novel questions concerning how information about ASR variation is perceived. Over which social neighborhood is this knowledge sampled—an individual’s own group, all of its neighboring groups, or even beyond? Furthermore, is this knowledge accumulated over time or can it be immediately assessed? Specific answers to these questions likely depend on the predominant species-specific modalities used for communication. In humans, information about sex ratios can be deduced from visual and acoustic stimuli that feed into an evolved psychological mechanism for functional, fast, and relatively automatic abilities to track local sex ratios^144,145^. These cognitive mechanisms to detect ASR variation remain unstudied in animals. Nonetheless, much of the currently available evidence indicates that individuals respond flexibly to their locally perceived ASR^146,147^. Natural, intraspecific fluctuations in ASR impact mate choice and breeding system, and potentially any frequency dependent behavioral and life-history strategy between the sexes. The limits of this plasticity remain currently unknown for most species; however, experimental manipulations of ASR in both vertebrates and invertebrates have yielded the strongest evidence for a causal role of ASR in adaptively shaping plasticity in sex role behaviors^91,94,101,111,148,149^.

### Population scale

Local ASRs may vary considerably—both spatially across populations—and temporally from year to year within a group. For example, humans historically lived in small populations, which are particularly susceptible to random variation in sex-biased births and deaths^12,150–152^. Hunter-gatherer groups are typically composed of 35-80 individuals, where, by chance, births may be predominantly male in one year and female in the next. A longitudinal study of neotropical hunter-gatherers found that in some years men outnumbered women by fourfold, while at other times the excess of women was nearly as extreme^12^. This demographic characteristic of small populations has direct implications for mating options and partner availability.

Scaling up from small- to large-scale societies, and from subsistence to market economies, deviations from sex-ratio parity are common. While the world-wide ASR hovers near an even number of men to women, nation states express wide variation in ASRs. Skewed ASRs today are caused by a number of demographically and behaviorally mediated factors, the most influential being son preference and economic migration^153–155^. Son preference, access to sex-selective abortion, female infanticide, and neglect of female health contribute to differential child mortality, and results in an excess of males during crucial reproductive years (Fig. 2c). Economic and labor migration across borders where males or females differentially relocate for work also influence nationally skewed ASRs. The latter trend is more common among men. In countries such as Bahrain, Oman, United Arab Emirates, and Qatar, men outnumber women by 2- or 3-fold^156^.

Female-biased abortion as a result of son preference has created highly skewed sex ratios in large parts of China and India. Although the sex ratio at birth has become somewhat less male-biased across the 2010s in both countries, males born at the peak of the sex ratio at birth (from 2000 to 2010) are now at or reaching reproductive age. In some areas of rural China, the excess of young men has resulted in ASRs approaching 60% male^156^. More numerous, but less extreme, are female-biased nations, including Nepal, which has the lowest global ASR of 44% male due to higher rates of male mortality and out-migration. The ASR in the EU, Canada and U.S. all hover near parity, although local ASRs can vary substantially^157–159^.

### Consequences of skew for human societies

Frequency-dependent mating and parenting decisions, and the concept of mating markets, apply equally well for human and non-human societies^5,54^, although human studies often report more subtle associations between ASR and social behavior than animal studies. For example, imbalanced ASRs are associated with rates of violence (Box 5)^158^, personality shifts^160^, socio-sexual orientation^161^, economic decision-making^145^, and intergroup relationships^12^. Furthermore, ASR predicts the formation and stability of pair bonds in human populations^157,162,163^. In female-biased communities, males tend to pursue short-term mating goals^161^. For example, in urban areas where ASRs are female-biased due to, in part, high rates of male incarceration and mortality, men have higher rates of sexual concurrency, and nonmarital fertility and single motherhood are at their highest. One consequence of this is that HIV transmission rates are higher in female-biased communities^159^.

In male-biased populations, on the contrary, men are more likely be married, part of a family, and sexually committed to one partner^157^. However, where men are abundant, many of them are unable to marry, and this is of particular concern—especially in societies where marriage is expected and is the primary path to pair-bonding. For example, in China, never-married men (termed ‘bare branches’) are at greater risk for depression and suicide^4^ and have a tendency toward antisocial behavior and violence. Together, this has raised concerns related to local societal stability and security. In both China and India, an excess of males appears to have contributed to sex industry expansion, female coercion, and bride trafficking^156^.

As in animal societies, male-biased populations can provide advantages to females, especially in societies where women have traditionally held low status^156^. Over time, the social position of women has increased in some male-biased societies, whereby women benefited from their enhanced standing by way of rarity, leading to more educational and economic opportunities as well as improved mental health outcomes^164^. For example, the relationship between the ‘value’ of women and their scarcity has contributed to increases in the proportion of female university graduates and female participation in the labor force in China. As of 2018, 52.5% of all undergraduate and 49.6% of all graduate students were women, and women made-up 43.7% of the total labor force—a striking transition in just a few generations^165^. In addition, monogamy is more prevalent in male-biased societies and women generally prefer long-term monogamous relationships compared to men^166^. This preference has been argued to explain the lower rates of premarital and extramarital sex and lower divorce rates in male-biased ASRs^167^. Finally, in male-biased societies women also have greater opportunity to marry-up with men of higher socio-economic status^155^. Ultimately, such material and social improvements for women have contributed to more balanced sex ratios at birth through a decline in son preference.

Box 5 More men = more violence? The association between ASR and male violence has been intensely studied in humans. The traditional argument purports that a surplus of men in a given population causes more men to be unpartnered, which leads them to compete more vigorously for mates^11,21,90^. Such competition sometimes occurs through violence, as evidenced by the observation that single men are overrepresented as violent offenders^192^. However, theoretical advancements have questioned this assertion and instead proposed that men will reduce mating effort when they are plentiful^20^, resulting in less violence. Male strategies of displaying and adhering to characteristics that women seek in long-term committed relationships may instead be favored. Studies on both human and nonhuman animals have shown that male reproductive skew is higher where females are in excess^5,7^. Yet, studies that evaluate the competing predictions have failed to provide convincing support for either side^5^, since the adult sex ratio has been both positively^193–199^ and negatively^158,200–203^ associated with violent crime, homicide, and sexual assault.

There are several potential reasons for these diverging results. First, there is considerable variation in the nature and quality of the data on violence employed. Violence is a sensitive topic; scholars are dependent on events that appear in registers or other data sources and the ecological fallacy is often an issue (i.e., making inferences about an individual based on aggregate data for a group)^204^. Second, it is not always possible to distinguish between male violence towards other men and towards women. Such a distinction is crucial as one might otherwise conflate intra- and intersexual competition. Third, mating effort will not necessarily involve violent behavior, nor is all violent behavior mating effort. With regards to the latter, other underlying factors, such as substance abuse or economic inequality may explain different rates of violence within and between populations. Whether an individual will resort to violence in the quest for mates might depend on the particular sociocultural context, as well as individual characteristics. Violence is unlikely to show a significant association with the ASR if some men pursue non-violent strategies to acquire a mate. Furthermore, only a few studies have examined whether men who have children or vary in socioeconomic status respond differently to a partner scarcity, but such studies suggest that differences do exist^199,205^.

In sum, while the human literature on sex ratio skew appears to show a fairly coherent picture for outcomes such as fertility and family formation, the impact on violence remains unresolved^206^. Given the potential direct and indirect effects on various societal issues, especially in large populations such as China and India, it is crucial to bring more clarity to this question. For instance, “tough on crime” policies that favor incarcerations may potentially exacerbate, rather than lessen, existing levels of violence^197^.

**Box 5 Fig: Weighing the evidence.** The empirical evidence for how adult sex ratios are linked to human mating behavior is mixed. However, on balance, studies from different contexts suggest that the prevalence of female-headed households and sexual risk-taking are higher under female-biased sex ratios, whereas marriage rates, fertility, and relationship stability are higher under male-biased sex ratios. For the outcome of male violence, the jury is still out as studies have demonstrated both positive and negative associations with ASR. Various factors, from data biases to lack of theoretical clarity, may be responsible for the contradicting patterns and need to be addressed in future work.
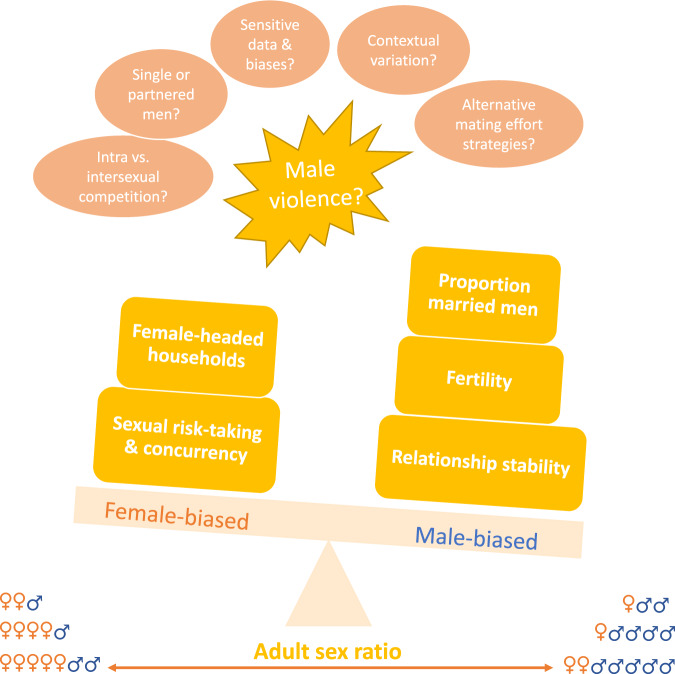

### Outlook

While both the causes and effects of variation in offspring sex ratio have been thoroughly explored over the past century, the sex ratio of adults has received far less attention. Recent studies of adult sex ratios are bringing together appealing features from different fields including anthropology, conservation biology, demography, behavioral ecology, and population dynamics. Combining these fields into a single framework to understand sex ratios produces unique and synergistic opportunities for the social and biological sciences. The way forward has been cleared by the many recent experimental and comparative analyses across animal taxa, and these studies attest to the novel insights that ASR-focused research can bring to social behavior in both human and non-human animal societies. Exploring the varied future prospects, through a multidisciplinary lens, will serve to both establish the importance of ASR across diverse fields and inform applied work and social policy on topics ranging from biodiversity conservation to public health.

### Reporting summary

Further information on research design is available in the Nature Research Reporting Summary linked to this article.

## Supplementary information

Reporting Summary-New
