# Author Correction: Adult sex ratios: causes of variation and implications for animal and human societies

**DOI:** 10.1038/s42003-022-04296-7

**Published:** 2022-12-07

**Authors:** Ryan Schacht, Steven R. Beissinger, Claus Wedekind, Michael D. Jennions, Benjamin Geffroy, András Liker, Peter M. Kappeler, Franz J. Weissing, Karen L. Kramer, Therese Hesketh, Jérôme Boissier, Caroline Uggla, Mike Hollingshaus, Tamás Székely

**Affiliations:** 1 Department of Anthropology, East Carolina University, Greenville, NC USA; 2 Department of Environmental Science, Policy and Management and Museum of Vertebrate Zoology, University of California, Berkeley, CA 94720 USA; 3 Department of Ecology and Evolution, University of Lausanne, 1015 Lausanne, Switzerland; 4 Ecology & Evolution, Research School of Biology, The Australian National University, Acton, Canberra 2601 Australia; 5 MARBEC Univ Montpellier, CNRS, Ifremer, IRD, Montpellier, France; 6 ELKH-PE Evolutionary Ecology Research Group, University of Pannonia, 8210 Veszprém, Hungary; 7 Behavioural Ecology Research Group, Center for Natural Sciences, University of Pannonia, 8210 Veszprém, Hungary; 8 Behavioral Ecology and Sociobiology Unit, German Primate Center, Leibniz Institute of Primate Biology, 37077 Göttingen, Germany; 9 Department of Sociobiology/Anthropology, University of Göttingen, 37077 Göttingen, Germany; 10 Groningen Institute for Evolutionary Life Sciences, University of Groningen, 9747 AG Groningen, The Netherlands; 11 Department of Anthropology, University of Utah, Salt Lake City, UT USA; 12 Institute of Global Health, University College London, London, UK; 13 Centre for Global Health, Zhejiang University School of Medicine, Hangzhou, P.R. China; 14 IHPE Univ Perpignan Via Domitia, CNRS, Ifremer, Univ Montpellier, Perpignan, France; 15 Stockholm University Demography Unit, Sociology Department, Stockholm University, 106 91 Stockholm, Sweden; 16 Kem C. Gardner Policy Institute, David Eccles School of Business, University of Utah, Salt Lake City, UT USA; 17 Milner Centre for Evolution, University of Bath, Bath, BA2 7AY UK; 18 ELKH-DE Reproductive Strategies Research Group, Department of Zoology and Human Biology, University of Debrecen, H-4032 Debrecen, Hungary

**Keywords:** Behavioural ecology, Social evolution

Correction to: *Communications Biology* 10.1038/s42003-022-04223-w, published online 19 November 2022.

The original version of this article contained errors in the text of Box 5, Box 1 and reference 5.

In Box 5, the text incorrectly stated: ..“men who have children or socioeconomic status”. It has been corrected to “men who have children or vary in socioeconomic status”.

In Box 1, “Du Bois’” has been corrected to “Du Bois”.

In Reference 5, the text “human societies that sets the agenda how reformulated” has been corrected to “human societies that sets the agenda for how reformulated”.

